# Health-Risk Behaviors and Dietary Patterns Among Jordanian College Students: A Pilot Study

**DOI:** 10.3389/fnut.2021.632035

**Published:** 2021-05-14

**Authors:** Hana Alkhalidy, Aliaa Orabi, Tamara Alzboun, Khadeejah Alnaser, Islam Al-Shami, Nahla Al-Bayyari

**Affiliations:** ^1^Department of Nutrition and Food Technology, Faculty of Agriculture, Jordan University of Science and Technology, Irbid, Jordan; ^2^Department of Clinical Nutrition and Dietetics, Faculty of Allied Health Science, The Hashemite University, Zarqa, Jordan; ^3^Department of Nutrition and Food Technology, Faculty of Al-Huson University College, Al-Balqa Applied University, Al-Salt, Jordan

**Keywords:** health risk, college students, dietary pattern, smoking, fast-food, behavior

## Abstract

**Background/Aims:** Health promotion and the incorporation of health-protective behaviors in people's lifestyles have a great role in enhancing individuals' overall health and well-being. College students are at increased risk of developing unhealthy dietary and lifestyle behaviors. A cross-sectional pilot study was conducted to assess the health-risk behaviors among undergraduate college students at Jordan University of Science and Technology.

**Methods:** The final sample included 136 students, with a mean age of 21.1 ± 2.37 years, mostly females (69%). A self-reported questionnaire was used for data collection about dietary and lifestyle behaviors among college students. The questionnaire consisted of four parts: sociodemographic characteristics, body weight classifications, lifestyle behaviors, and dietary patterns and intake, and eating behaviors.

**Results:** Most of the students did not meet the daily recommendations for fruit (76%) and vegetable (82%) intake. Males were significantly consuming fast food more frequently (*p* = 0.019), and smoked cigarettes (*p* < 0.001) or hookah (*p* = 0.015) more frequently than did females. Further, the majority met the recommendations for physical activity (81%), but exceeded recommendations for sedentary behavior. Females were more likely to have normal weight or be underweight (OR = 4.865), to have a fear of weight gain (OR = 3.387), and to have the recommended sleeping hours (OR = 7.685) than were males.

**Conclusion:** The results indicate the health-risk behaviors and the gender-related differences among college students.

## Introduction

Nowadays, the world faces a double burden of communicable and non-communicable diseases (NCDs), with a higher threat in developing countries and a higher incidence of premature deaths caused by NCDs (1). Adding to this the ancillary burden of the coexistence of undernutrition and overnutrition (2). Overnutrition, including overweight and obesity, is identified as one metabolic risk factor among other modifiable risky behaviors that increase the risk of developing NCDs (3). Hence, health promotion is a crucial strategy in reducing the worldwide burden of diseases by addressing and controlling behavioral risk factors among whole communities and the populations at high risk of adverse health outcomes (4). Several behavioral risk factors affect different aspects of individuals' health, and collectively they produce the person's lifestyle (5).

Lifestyle behaviors are classified as health-protective or health-risk behaviors. Health-protective lifestyle behaviors minimize disease risk or restore overall health (6). Examples include healthy nutrition, physical activity, stress management, smoking avoidance, and restful sleep (7, 8). Long-term adherence to these behaviors is necessary for preventing chronic diseases and reducing disease-related mortality (9, 10). Health-risk behaviors are frequently adopted activities that increase the risk of disease or injury (11), such as smoking, sedentary lifestyle, and inadequate intake of fruits and vegetables (12). These health risk behaviors and other NCDs risk factors such as obesity are modifiable factors, unlike age, gender, and family history that are considered non-modifiable factors (13). The clustering of modifiable risk factors related to chronic diseases might exaggerate the burden of disease and NCDs-related mortality (14, 15). Health-risk behaviors are the primary factors leading to NCDs, which account for 71% (41 million) of global deaths, including premature deaths (15 million) (3).

One of the primary targets in the United Nations' agenda for sustainable development is to reduce premature deaths due to NCDs by one-third by 2030 through prevention, treatment, and health promotion (16). According to the World Health Organization, this target could be achieved by modifying the four main behaviors that contribute to NCDs: tobacco use, unhealthy diet, physical inactivity, and harmful alcohol use (3, 17). Both physical inactivity and sedentary behaviors might increase the risk of developing some NCDs such as cardiovascular diseases (CVDs) and type 2 diabetes (T2D) (18). Short sleep duration is another risky behavior strongly associated with an increased risk of CVDs and T2D (19). Health-risk behaviors were seen among young adults, including college students, thus increasing their risk for developing chronic diseases (20, 21). These behaviors might be exaggerated by university life, environmental factors, and students' socioeconomic status (22, 23). Hence, assessing these behaviors is necessary to develop an effective future intervention for managing these factors and preventing the occurrence of NCDs. This study aims to assess the health-risk behaviors among undergraduate college students at Jordan University of Science and Technology.

## Materials and Methods

### Study Design and Participants

A cross-sectional pilot study was carried out between June and October 2019 at Jordan University of Science and Technology (JUST) in Irbid, Jordan. Jordanian undergraduate students aged above 18 years, regardless of their gender and academic specialty, had the chance to take part in the study. Pregnant students, graduate students, and non-Jordanians were excluded from the study. The final sample comprised 136 students. The study protocol was approved by the Institutional Review Board at JUST. All participants signed an approved consent form. The researcher clearly explained the study's purpose and procedure, informed the participants about their withdrawal rights, and informed participants of confidentiality in handling information.

### Data Collection Procedure

A self-reported questionnaire was used in data collection. The study questionnaire was developed based on a thorough literature review and revised by the research team, experienced professionals, and experts in the field. All questions were assessed for their clarity, consistency, and relevance to the study aims. The questionnaire was pilot tested on a sample of college students (*n* = 25). The students were informed about the study objectives and guided on how to complete the questionnaire. A 10-min discussion session was conducted after the completion of the questionnaire to discuss the questionnaire's clarity and modifications proposed by the students. The final revised questionnaire consisted of multiple-choice and fill-in-the-blank questions, and comprised four parts (sociodemographic characteristics, body weight classifications, lifestyle behaviors, and dietary patterns and intake, and eating behaviors) (Supplementary Data).

Anthropometric measurements, including weight and height, were measured using a digital electronic scale (body fat scale GW22029, GoWISE USA, U.S.) and a stadiometer (portable mechanical stadiometer HM200P, Charder, Taiwan). Body mass index (BMI) was calculated according to Quetelet's formula: [weight (kg)/height^2^ (m^2^)] (24) and classified according to world health organization criteria (BMI was categorized as: Underweight <18.50, normal 18.5–24.99, overweight 25.0–29.99, and obese ≥ 30.0) (25). The student's perceived weight, as well as mother's and father's weight status, was determined using Stunkard's figure rating scale. Stunkard's Scale comprises nine shape figures that increase in size from very thin to very obese (26).

Students' physical activity was assessed by self-reporting light, moderate, and vigorous physical activities (per day, week, month, and 6 months). Students met the requirements if they reported 150 min of moderate or 75 min of vigorous-intensity physical activity weekly, or an equivalent combination thereof (18). Sedentary behaviors, including sitting for > 4 h per day (27), and using screens for > 2 h per day (28, 29), were contributors to increased health risks among the students. Sleep duration was classified according to the national sleep foundation. The students met the recommendations with 7–9 h of sleep, and had an acceptable amount of sleep with 6 or 10–11 sleeping hours (30).

Dietary information was gathered using questions about dietary pattern, dietary intake, and eating behaviors. A food frequency questionnaire describing fruits and vegetables intake was also used. Students were classified according to their fast food intake, using values of ≤ 1 time per week, or > 1 time per week (31). Further, students met recommendations with daily consumption of 4 servings of fruit, and 5 servings of vegetables for females or 6 for males (32).

### Statistical Analysis

Analyses were carried out using SPSS software (IBM SPSS Statistics for Windows, Version 21.0. Armonk, NY: IBM Corp, *SCR_019096*). Continuous variables were described using means and standard deviations, and categorical variables were described using percentages. The Chi-square test was used to compare percentages. Univariate and multivariate logistic regression analyses were performed to examine the determinants of obesity among the study population, including sociodemographic, lifestyle, eating behaviors, dietary intake and pattern. For each variable, the number of non-missing values was used. We specified the missing = listwise sub-command to exclude data if a value is missing for any variable in the list. A *P* < 0.05 was considered statistically significant.

## Results

### Sociodemographic and Lifestyle Characteristics

A total of 136 students participated in this study; their mean age was 21.1 ± 2.37 years. As presented in Table 1, most of the students were females (69%), single (95%), more than half (54%) lived in an urban area and had a family income between 350 and 799 JOD (57%).

**Table 1:** The sociodemographic characteristics of the study population stratified by gender (*n* = 136).

**Variable**	**Total n (%)**	**Males (*n* = 42)**	**Females (*n* = 94)**	***P-*value^b^**
**Age group^a^**				
≤ 20 years	66 (49)	21 (51)	45 (48)	0.721
> 20 years	69 (51)	20 (49)	49 (52)	
**Marital status**				
Single	129 (95)	42 (100)	87 (93)	0.069
Married	7 (5)	0 (0)	7 (7)	
**Living place**				
Urban	74 (54)	19 (45)	55 (59)	0.151
Rural	62 (46)	23 (55)	39 (41)	
**Family income (JOD)**				
<350	14 (10.3)	5 (11.9)	9 (9.6)	0.731
350–799	78 (57.4)	22 (52.4)	56 (59.6)	
800 or more	44 (32.4)	15 (35.7)	29 (30.9)	
**Family size**				
≤ 1 sibling	15 (11)	3 (7)	12 (13)	0.333
> 1 sibling	121 (89)	39 (93)	82 (87)	

*JOD, Jordanian Dinar*.

a *The total does not equal 136 as the age (n = 135) was missing for one male participant*.

b *P <0.05 was considered significant for Chi-square test*.

**Figure 1:**
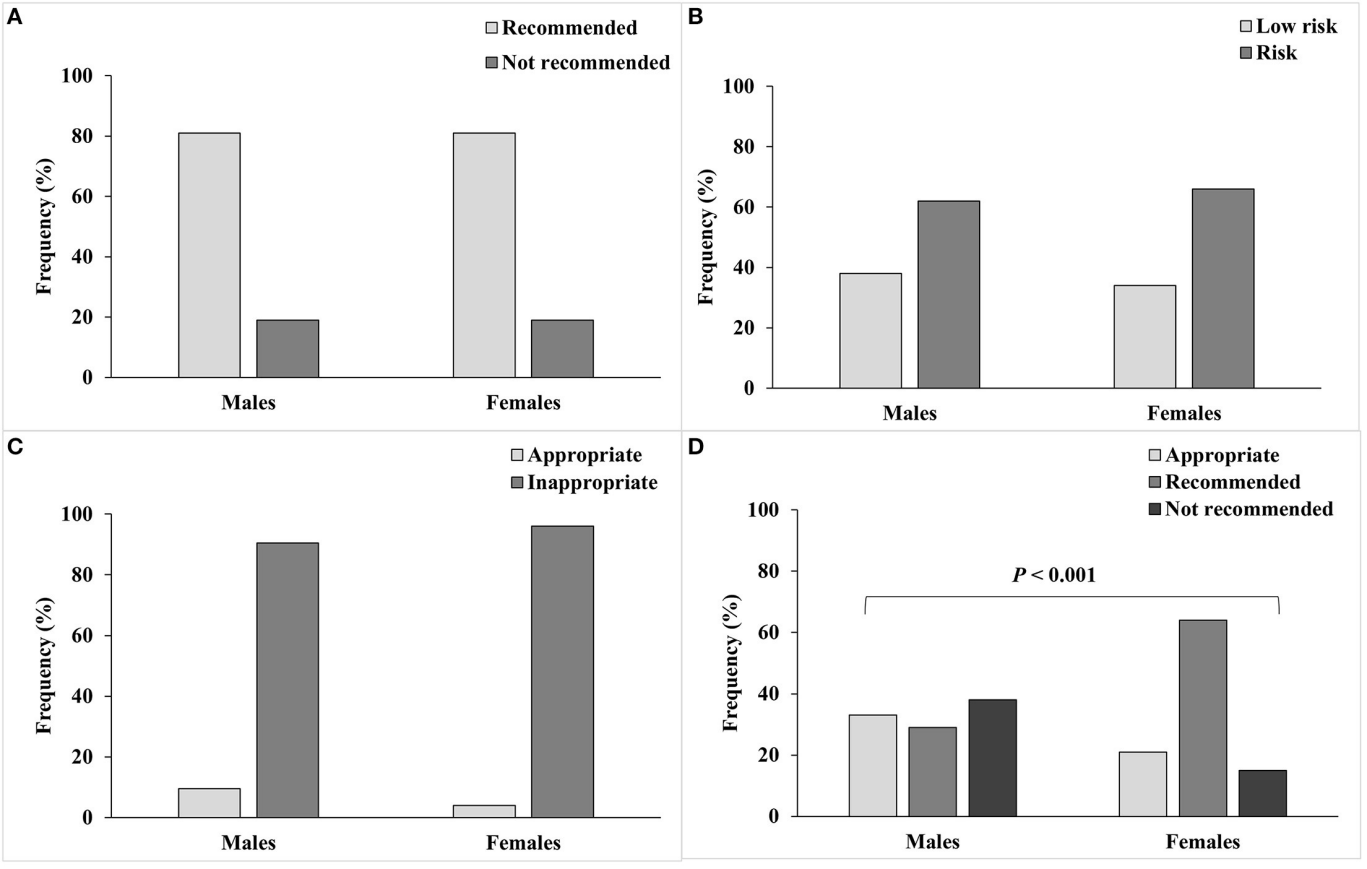
Lifestyle behaviors of male and female college students and their classification according to reference recommendations. **(A)** Physical activity; 150 min of moderate intensity or 75 min of vigorous intensity activities, or an equivalent combination is recommended, **(B)** Hours sitting; sitting ≤ 4 h per day was considered low risk, **(C)** Screen use; screen time of ≤ 2 h per day was considered appropriate, and **(D)** Sleeping hours; 7–9 h of sleep is recommended, and 6 or 10–11 h of sleep is appropriate.

As shown in Figure 1, ~81% of the students were physically active (Figure 1A), 65% displayed increased time sitting (Figure 1B), and 94% displayed risky screen use (Figure 1C). Our study showed that 22% of students were not getting enough sleep, which was higher in males (38%) compared to females (15%) (*P* < 0.001) (Figure 1D).

**Figure 2:**
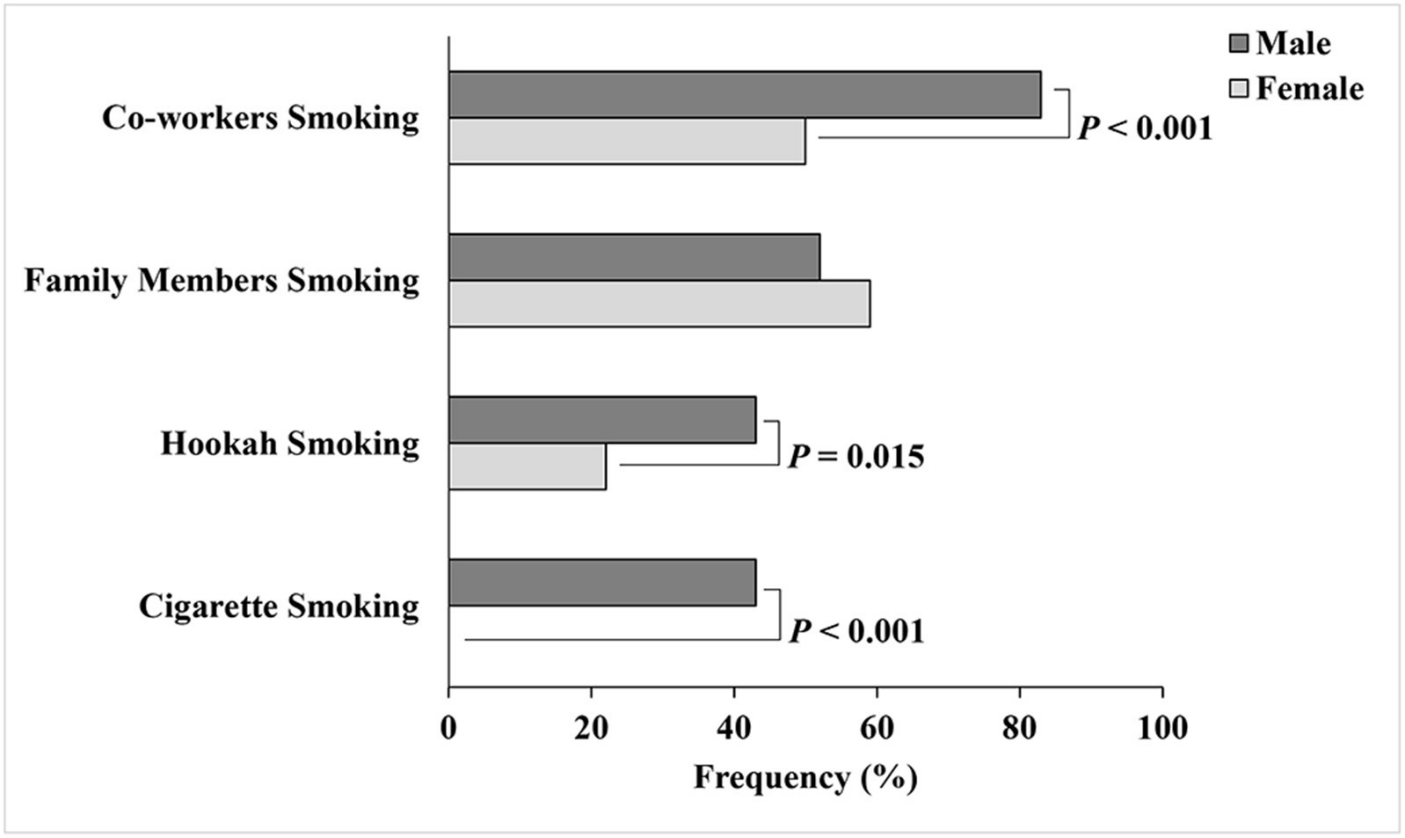
Smoking status of male and female college students, family members, and co-workers.

Figure 2 presents the smoking status of the students, their families, and co-workers. About 13% of the students smoked cigarettes and 29% smoked hookah. All females in our study were non-cigarette smokers; however, 22% were hookah smokers. The percentages of cigarette (*P* < 0.001) and hookah (*P* = 0.015) smoking were significantly higher among males than among females. More than half of the students had a family member who smoked (57%) or a co-worker who smoked (60%). Also, more males (83%) reported smokers' presence in the working place than did females (50%) (*P* < 0.001).

### Anthropometric Measurements and Eating Behaviors

**Figure 3:**
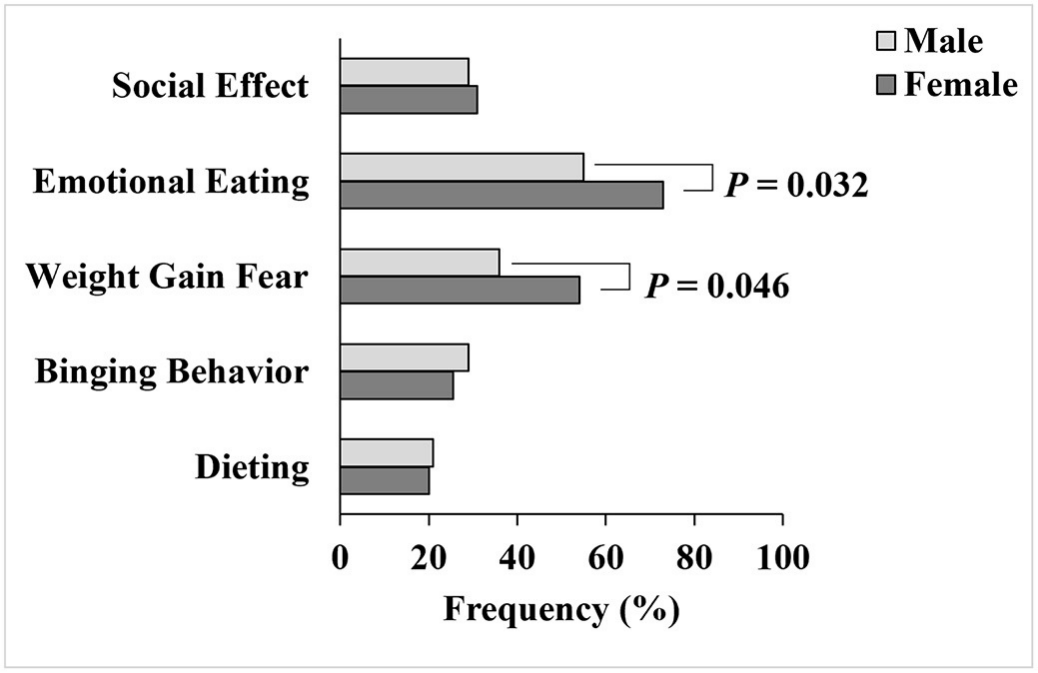
Dieting and eating behaviors of male and female college students.

As shown in Table 2, most students failed to describe their actual body weight (72%) in our study. The measured BMI differed significantly between male and female students (*P* = 0.012). Males were more overweight and obese than females (37 vs. 17%), while females were more normal or underweight than males (83 vs. 63%) (Table 2). The results showed that 48.5% of the students had a concern about weight gain and 68% of them had emotional eating behavior. Female students were more concerned about their body weight (54 vs. 36%, *P* = 0.046) and more prone to emotional eating behavior than males (73 vs. 55%, *P* = 0.032). Other eating behaviors, including dieting (21%), binge eating (27%), and altered eating habits related to social pressure (30%), were present among the students (Figure 3).

**Table 2:** Body weight status stratified by gender (*n* = 136).

**Variable**	**Total n (%)**	**Males**	**Females**	***P*-value^b^**
		**(*n* = 42)**	**(*n* = 94)**	
**BMI classification^a^**				
<25	101 (77)	26 (63)	75 (83)	0.012
≥ 25	30 (23)	15 (37)	15 (17)	
**Described body weight^c^**				
Exact BMI estimate	37 (28)	8 (19.5)	29 (32)	0.134
Different BMI estimate	94 (72)	33 (80.5)	61 (68)	
**Described mother weight**				
Underweight	20 (15)	3 (7)	17 (18)	0.212
Normal	79 (58)	28 (67)	51 (54)	
Overweight	34 (25)	11 (26)	23 (25)	
Obese	3 (2)	0 (0)	3 (3)	
**Described father weight**				
Underweight	23 (17)	10 (23.8)	13 (14)	0.346
Normal	61 (45)	17 (40.5)	44 (47)	
Overweight	49 (36)	15 (35.7)	34 (36)	
Obese	3 (2)	0 (0.00)	3 (3)	

a *BMI, Body Mass Index. Classified in 2 groups: <25 indicate normal and underweight; ≥ 25 indicate overweight and obesity*.

b *P <0.05 was considered significant for Chi-square test*.

c *The total does not equal 136 due to the missing data by participants according to described body weight (n = 131)*.

### Dietary Intake and Pattern

**Figure 4:**
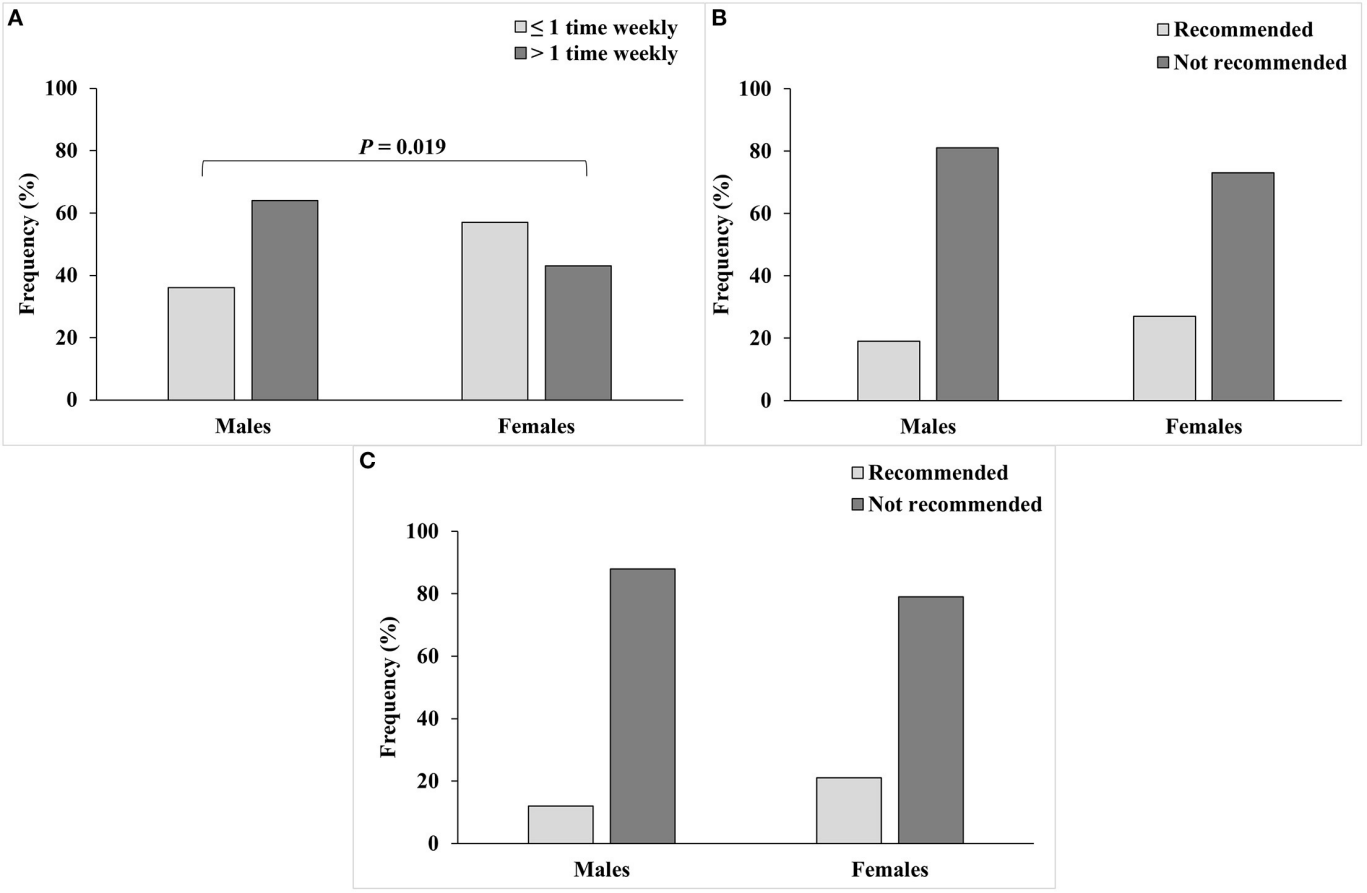
Dietary intake for male and female college students from **(A)** Fast food, **(B)** Fruits; 4 servings per day for both genders is recommended, and **(C)** Vegetables; 5 servings per day for females and 6 servings per day for males are recommended.

The dietary pattern of university students is shown in Table 3. Nearly half of the students had two main meals per day, 67% had breakfast daily, and 63% had less than or equal to two snacks per day. The analysis of the dietary intake showed that 49% of the students consumed fast food more than once weekly, a value that was higher in males than females (64 vs. 43%, *P* = 0.019) (Figure 4A). Most students did not meet the daily recommendations for intake of fruits and vegetables (76 and 82%), the insufficiency being insignificantly higher in males compared to females (Figures 4B,C, respectively).

**Table 3:** Dietary intake and pattern of college students stratified by gender (*n* = 136).

**Variable**	**Total n (%)**	**Males (*n* = 42)**	**Females (*n* = 94)**	***P*-value^a^**
**Number of meals**				
1 Meal	18 (13)	6 (14.3)	12 (13)	0.254
2 Meals	69 (51)	17 (40.5)	52 (55)	
≥ 3 Meals	49 (36)	19 (45.2)	30 (32)	
**Number of snacks**				
≤ 2 Snacks	86 (63)	25 (59.5)	61 (65)	0.548
> 2 Snacks	50 (37)	17 (40.5)	33 (35)	
**Having breakfast**				
Yes	91 (67)	27 (64)	64 (68)	0.664
No	45 (33)	15 (36)	30 (32)	
**Fast food eating**				
Yes	114 (84)	32 (76)	82 (87)	0.106
No	22 (16)	10 (24)	12 (13)	
**Dietary supplement use**				
Yes	13 (10)	1 (2)	12 (13)	0.057
No	123 (90)	41 (98)	82 (87)	

a *P <0.05 was considered significant for Chi-square test*.

### Univariate and Multivariate Logistic Regression Analyses

Table 4 represents the adjusted association between sociodemographic characteristics, lifestyle behaviors, eating behaviors, dietary intake and pattern, and weight status with gender by using the stepwise selection method, with entry testing based on the significance of the score and removal testing based on the probability of a likelihood-ratio based on the conditional parameter estimates. Due to the absence of married males and cigarette-smoking females, marital status and cigarette smoking were excluded from the regression model. All observed findings showed no statistically significant differences across both genders except for the living place, co-workers smoking, sleeping hours, BMI classification, and having fear about weight gain. Therefore, these variables were included in the last step of the regression model. Findings showed that females were three times more likely to live in urban areas than males (*P* = 0.020), while they were less likely to suffer from second-hand smoke from co-workers (adjusted odds ratio = 0.125; *P* = 0.001) compared to males. Further, the regression equation revealed that females were about eight times more likely to reach the recommended hours of sleep (*P* = 0.001). Finally, females were three times more likely to have concerns about weight gain (*P* = 0.018) and almost five times more likely to be classified as normal or underweight (*P* = 0.005) when compared to males.

**Table 4:** Logistic regression model for the study population according to gender.

**Variable^‡^**	**OR^a^**	**95% CI^a^**	***p*-value^b^**
**Living place**				
Urban	3.348	1.206–9.292	0.020
Rural	*Reference*		
**Co-workers smoking**				
Yes	0.125	0.041–0.384	0.001
No	*Reference*		
**Sleeping hours**				
Recommended	7.685	2.814–20.986	0.001
Not recommended	*Reference*		
**BMI classification**^a^				
<25	4.865	1.602–14.772	0.005
≥ 25	*Reference*		
**Weight gain fear**				
Yes	3.387	1.229–9.330	0.018
No	*Reference*		

‡ *Presented data are adjusted for all other variables in the study*.

a *OR, odds ratio; CI, confidence interval; BMI, Body Mass Index*.

b *P <0.05 was considered significant*.

## Discussion

This pilot study aimed to assess health-risk behaviors among college students. Most participants in this study sample were physically active, which is consistent with the college students in countries such as Ireland (92% met the recommendations) (33), and contrary to students in countries such as Canada (65% were sedentary) (34). Although most of the students in our study met the physical activity recommendations, they were at increased health risks because of increased sitting time, which corresponded to students behavior among a sample of U.S. college students (35). Prolonged sitting was one of the sedentary behaviors associated with an increased risk of developing chronic conditions like CVDs, obesity, T2D, metabolic syndrome, and cancer (36). Nearly all students used screens for more than 2 h, which is higher than the recommended time. Similarly, Abu-Mweis et al. found that 95% of Jordanian adolescents spent more than 2 h on screens (37). Screen time is a recognized health-risk behavior, as previous studies suggested that exceeding 2 h daily of screen use might be associated with adverse physiological and psychological health effects such as obesity, anxiety, and depression (38–40).

Having enough sleep is necessary to avoid sleep deprivation consequences including fatigue, decreased well-being, daytime dysfunction, decreased academic performance, and decreased learning abilities (41). Cassidy et al. reported an association between NCDs, including CVDs and T2D, and not meeting recommendations for hours of sleep (42). Our study showed a significant gender difference, as females were about eight times more likely to achieve the recommended hours of sleep compared to males. Our results were consistent with previous studies as females had a longer sleep duration and a lower risk of inadequate sleep than males (43, 44). However, in China, female students had lower sleep duration and quality than males (45). Evidence supported the correlation between short sleep duration and increased risk of smoking, drinking alcohol, risky eating behaviors, mental issues, sexual activity, and illicit drug use (36, 46, 47).

Smoking was higher among male participants compared to females in our study which corresponds to findings of a Turkish study wherein males were three times more likely to be smokers than females (48). Our findings might be related to the cultural acceptance of smoking among males but not among females (49). However, hookah smoking might be more acceptable among females in the Arab region (50), explaining hookah smoking, but cigarette smoking, by female students in our study. Nonetheless, cigarette smoking among Arab females might be underreported due to cultural constrains (51), perhaps explaining the absence of cigarette smoking in our female participants. Smoking, in all its forms, might increase the students' risk of developing NCDs, including CVDs, respiratory diseases, and cancers (52). Our study also showed an increased presence of smokers in the males' workplaces compared to those of females. Hence, this might contribute to the higher percentage of smoking among males than females, as the individual's smoking behavior is affected by the social environment, including behaviors of friends and co-workers (53, 54).

Most students, in our study had an inaccurate BMI perception. In comparison, few college students in the U.S. (28%) and the United Arab Emirates (14%) perceived their weight inaccurately (55, 56). Weight perception might be associated with eating behaviors, or with higher weight perception indicating increased eating disturbances (57). These disturbances are linked with several chronic conditions including T2D and hypertension (58). In our sample, overweight and obesity percentages were lower than in a previous study conducted on university students in Jordan. However, Amr et al. reported that more males were overweight and obese than females (59), which was consistent with our findings as females were five times more likely to be classified as normal or underweight when compared to males. These findings might be related to an increase in the fear of weight gain among our female subjects (60). Further, females might have a higher level of emotional eating compared to males (61). The higher prevalence of overweight and obesity among our male students might put them at an increased risk of developing NCDs and the related metabolic risk factors (62).

Most of our study sample consumed fewer than three meals a day. Likewise, in Iran, about half of the students had either one or two meals per day (63). Previous studies showed that skipping main meals might be associated with weight gain and obesity (64, 65). One-third of our participants did not have breakfast regularly. As shown in the literature, skipping breakfast was common among college students (63, 66). Mansouri et al. reported that regular breakfast consumption was inversely associated with overweight and obesity in college students (67). The students' higher tendency to skip meals might be associated with an increased risk of poor snacking behavior (68). Snacking between meals might be correlated with an increased risk of being overweight or obese (69). Our results showed that most of the students had less than two snacks per day, while about one-third of them had over two snacks per day, which was insignificantly higher among males. Similarly, in India, males had more frequent snacking behavior compared to females (68).

Several studies showed the inadequate daily consumption of fruits and vegetables among college students (63, 70, 71), with an increased intake of fast food (72, 73), which was also seen in this study. Our results were consistent with previous studies that revealed a higher fast-food intake among males and higher intake of fruits and vegetables among female college students (72, 74, 75). The consumption of fast food has been one of the major risk factors for obesity (76), while fruit and vegetable intake has been correlated with lower BMI in males and females (77). Hence, the dietary choices among our study population might explain, to some extent, the higher percentage of overweight and obesity in males compared to females in this study. The frequent intake of fast food might be associated with several metabolic risk factors such as increase blood pressure (78) and insulin resistance (79). Further, the inadequate fruit and vegetable intake was linked to increased insulin resistance (80), increased risk of NCDs, including CVDs and cancer (81), and increased risk of disease-related mortality (82). These poor dietary behaviors among college students may increase the risk of developing NCDs (83, 84), and understanding the overall dietary behaviors might be necessary to reduce this risk (85). Our college students might benefit from nutrition education programs to promote healthy food choices and provide adequate nutrition (86).

Our study might have some limitations, and the results should be interpreted with caution. The data was collected using self-reported and food frequency questionnaires, thus, there is a chance of under-reporting or over-reporting for the studied behaviors. Further, the sample was recruited from one large public university in Jordan, so the results cannot be generalized among all college students, and more research is needed to confirm our findings. However, our results provided evidence about several health-risk behaviors among the students. It also showed the importance of considering gender as a determining factor for these behaviors among college students, as some behaviors might be more prevalent in males than in females and vice versa. Further research is needed to highlight these differences and to identify the determinants of these behaviors.

## Data Availability Statement

The original contributions presented in the study are included in the article/Supplementary Material, further inquiries can be directed to the corresponding author/s.

## Ethics Statement

The studies involving human participants were reviewed and approved by Institutional Review Board at Jordan University of Science and Technology. The patients/participants provided their written informed consent to participate in this study.

## Author Contributions

HA study concept and design, interpretation of data, critical revision of the manuscript for important intellectual content, obtained funding, administrative, technical support, and study supervision. AO and TA conducted the study, data collection, and interpretation. KA and NA-B data interpretation and critical revision of the manuscript for important intellectual content. IA-S statistical analysis, interpretation of data, and critical revision of the manuscript for important intellectual content. All authors agree to be accountable for the content of the work.

## Conflict of Interest

The authors declare that the research was conducted in the absence of any commercial or financial relationships that could be construed as a potential conflict of interest.
