# Supplementary material for: Health-Risk Behaviors and Dietary Patterns Among Jordanian College Students: A Pilot Study
Source: Front Nutr. 2021 May 14;8:632035. doi: 10.3389/fnut.2021.632035 (PMC8160432; doi:10.3389/fnut.2021.632035)
Supplement: Supplementary file 1 [file Table_1.DOCX]

Supplementary Material

Questionnaire

**استبيان**

**رقم الاستبيان ( )**

**التاريخ:**

| **أولا: المعلومات الديموغرافية والاجتماعية والاقتصادية:** | | | | | | | | | | | | | | | | | |
| --- | --- | --- | --- | --- | --- | --- | --- | --- | --- | --- | --- | --- | --- | --- | --- | --- | --- |
|  | | | | | | | | | | | | | | | | | |
|  | | |  | | |  | | 1. **العمر: -----------** | | | | | | | | | |
|  | | | - أنثى | | | | | | | | | | - ذكر | | 1. **الجنس:** | | |
|  | | | | | | | | - مدينة | | | - قرية | | | | 1. **مكان السكن:** | | |
| - أرمل | | | | - متزوج | | | | - مطلق | | | - أعزب | | | | 1. **الحالة الاجتماعية:** | | |
| - أكثر من 800 | | | | - 350 - 800 | | | | - أقل من 350 | | | 1. **الدخل الشهري (دينار أردني):** | | | | | | |
| 1. **عدد أفراد الأسرة: -----------** | | | | | | | | | | | | | | | | | |
| **ثانيا: المعلومات الصحية:** | | | | | | | | | | | | | | | | | |
|  | | | | | | | | | 1. **الوزن: ----------- كجم** | | | | | | | | |
|  | | | | | | | | | 1. **الطول: ----------- سم** | | | | | | | | |
| 1. **من خلال الأشكال الموضحة أدناه قم بتحديد وزن الأم:** | | | | | | | | | | | | | | | | |  |
| 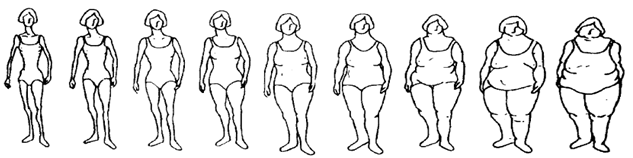 | | | | | | | | | | | | | | | | |  |
| - **1** | - **2** | - **3** | | | - **4** | | - **5** | | | - **6** | | - **7** | | - **8** | | - **9** |  |

| 1. **من خلال الأشكال الموضحة أدناه قم بتحديد وزن الأب:** | | | | | | | | |
| --- | --- | --- | --- | --- | --- | --- | --- | --- |
| 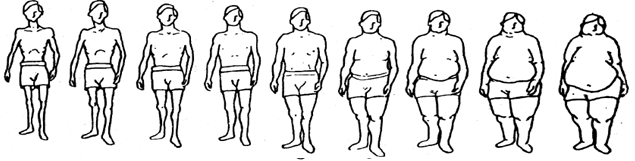 | | | | | | | | |
| - **1** | - **2** | - **3** | - **4** | - **5** | - **6** | - **7** | - **8** | - **9** |
| 1. كيف تقيم جسمك بناء على الأشكال التالية: | | | | | | | | |
| للإناث | | | | | | | | |
| 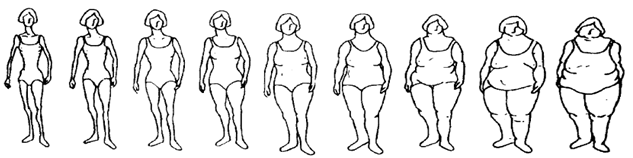 | | | | | | | | |
| - **1** | - **2** | - **3** | - **4** | - **5** | - **6** | - **7** | - **8** | - **9** |
| للذكور | | | | | | | | |
| 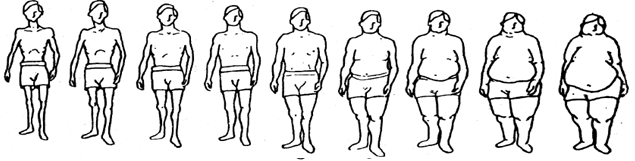 | | | | | | | | |
| - **1** | - **2** | - **3** | - **4** | - **5** | - **6** | - **7** | - **8** | - **9** |

| - لا | | | | - نعم | | | | | | 1. **هل تشعر بضغوط اجتماعية بالنسبة لوزنك؟** | | | | | |  |
| --- | --- | --- | --- | --- | --- | --- | --- | --- | --- | --- | --- | --- | --- | --- | --- | --- |
| - لا | | | | - نعم | | | | | | 1. **هل تشعر بالخوف من زيادة الوزن؟** | | | | | |  |
| **ثالثا: نمط الحياة والمعيشة:** | | | | | | | | | | | | | | | |  |
| 1. **التدخين:** | | | | | | | | | | | | | | | |  |
| - لا | | | - نعم | | | | | | | **هل أنت مدخن للسجائر؟** | | | | | |  |
| - لا | | | - نعم | | | | | | | **هل تستخدم الشيشة / الأرجيلة؟** | | | | | |  |
| - لا | | | - نعم | | | | | | | **هل يوجد مدخنين في العائلة؟** | | | | | |  |
| - لا | | | - نعم | | | | | | | **هل زملائك في مكان العمل مدخنين؟** | | | | | |  |
| 1. **املأ الجدول التالي بما يتناسب مع الوقت الذي تقضيه يوميا في كل من:** | | | | | | | | | | | | | | | |  |
|  | | | | | | **عدد ساعات استخدام الشاشة (مثل: التلفون، الكومبيوتر، ألعاب الفيديو)** | | | | | | | | | |  |
|  | | | | | | **عدد ساعات الجلوس** | | | | | | | | | |  |
|  | | | | | | **عدد ساعات النوم** | | | | | | | | | |  |
| 1. **النشاط البدني** | | | | | | | | | | | | | | | |  |
| **املأ الجدول بما يتناسب مع نشاطك الرياضي، يرجى الإجابة مع العلم أن:**   - الرياضة قليلة الشدة تشمل (كنس المنزل، غسيل الملابس يدويا، غسيل السيارة، رفع أشياء خفيفة، مشي بطيء) - الرياضة متوسطة الشدة تشمل (كره الطائرة، تنس الطاولة، ممارسة رياضة منزلية بجهد متوسط، مشي بسرعة عالية). - الرياضة مرتفعة الشدة تشمل (رفع أشياء ثقيلة، حرث الأرض، ركوب الدراجة، الجري، كرة القدم، كرة السلة، السباحة، نط الحبل). | | | | | | | | | | | | | | | |  |
| **دقيقة / ستة أشهر** | **دقيقة / الشهر** | | | **دقيقة / الأسبوع** | | | **دقيقة / اليوم** | | | | | **نوع الرياضة** | | | |  |
|  |  | | |  | | |  | | | | | **قليلة الشدة** | | | |  |
|  |  | | |  | | |  | | | | | **متوسطة الشدة** | | | |  |
|  |  | | |  | | |  | | | | | **مرتفعة الشدة** | | | |  |
| **رابعا: المعلومات الغذائية:** | | | | | | | | | | | | | | | | |
| 1. **كم عدد الوجبات الأساسية التي تتناولها باليوم؟** | | | | | | | | | | | | | | | | |
| - أكثر من ذلك | | | - ثلاث وجبات | | | | | - وجبتين | | | | | - وجبة واحدة | | | |
| 1. **كم عدد الوجبات الخفيفة التي تتناولها باليوم؟** | | | | | | | | | | | | | | | | |
| - أبدا | | - أكثر من ذلك | | | - ثلاث وجبات | | | | | | - وجبتين | | | - وجبة واحدة | | |
| - لا | | - نعم | | | | | | | | | | | 1. **هل تتناول وجبة الإفطار؟** | | | |
| - لا | | - نعم، كم مرة خلال الأسبوع أو الشهر **-----** | | | | | | | | | | | 1. **هل تتناول الوجبات السريعة؟** | | | |
| - لا | | - نعم | | | | | | | | | | | 1. **هل تتبع نظام غذائي؟** | | | |

| 1. **هل تستخدم أي من أنواع المكملات الغذائية؟** | | | | |
| --- | --- | --- | --- | --- |
| - لا | | - نعم | |  |
| 1. **هل تتأثر شهيتك والكميات المتناولة بالحالة النفسية؟** | | | | |
| - لا | | - نعم | |  |
| 1. **هل تقوم بتناول كميات كبيرة من الطعام بحيث تفقد السيطرة على كمية ونوعية الطعام المتناول وتعجز عن التوقف عن الأكل؟** | | | | |
| - لا | | - نعم | |  |
| 1. **هل تميل إلى تناول الطعام بعيدا عن أعين الآخرين، حتى إن لم تكن جائعا، بسرعة وبكميات كبيرة حتى تصل إلى شعور غير مريح بالامتلاء وعدم الرضا عن الذات؟** | | | | |
| - لا | | - نعم | |  |
| 1. **ماذا تفعل عندما تكثر من تناول الأغذية؟** | | | | |
| - لا شيء مما ذكر | - الصوم | | - الرياضة الشديدة | - الاستفراغ |

| **Food Frequency Questionnaire (short form)**  **استبيان قصير لتعدد استهلاك الخضار والفواكه: يرجى ملء الجدول التالي بما يتناسب مع الكمية المتناولة من الأطعمة التالية:** | | | | | | | | | | |  |
| --- | --- | --- | --- | --- | --- | --- | --- | --- | --- | --- | --- |
| **المجموعة الغذائية** | **يوميا** | **أسبوعيا** | | | **شهريا** | | **حجم الحصة المتوسطة** | **حجم الحصة** | | | |
|  |  | **5-6** | **3-4** | **1-2** | **1-3** | **أقل من 1 - أبدا** |  | **صغير** | **متوسط** | **كبير** | |
| **الفواكه** | | | | | | | | | | | |
| الفواكه الطازجة (مثال: البرتقال، الجريب فروت، اليوسفي، الليمون، التفاح، الكمثرى، الخوخ، البرقوق) |  |  |  |  |  |  | حبة واحدة متوسطة |  |  |  | |
| الفواكه الطازجة (مثال: الفراولة والتوت، والعنب، والكرز، الأناناس) |  |  |  |  |  |  | نصف كوب |  |  |  | |
| الموز |  |  |  |  |  |  | حبة واحدة متوسطة |  |  |  | |
| البطيخ، الشمام |  |  |  |  |  |  | شريحة واحدة متوسطة |  |  |  | |
| الفواكه المجففة (مثال: الزبيب والخوخ) |  |  |  |  |  |  | ربع كوب |  |  |  | |
| عصائر الفواكه غير المحلاة (مثال: كوكتيل، برتقال، تفاح، أناناس) |  |  |  |  |  |  | نصف كوب |  |  |  | |
| **الخضار** | | | | | | | | | | | |
| المطبوخة (مثال: خضار مشوية أو مسلوقة، ملوخية، بامية، كوسا، زهرة، بروكلي، باذنجان) |  |  |  |  |  |  | نصف كوب |  |  |  | |
| الخضار الطازجة غير المطبوخة (مثال: سلطة خضار، بندورة، خيار، جزر) |  |  |  |  |  |  | كوب واحد |  |  |  | |
| خضروات نشوية (مثال: ذرة، بطاطا، فاصولياء خضراء) |  |  |  |  |  |  | نصف كوب |  |  |  | |
| عصائر الخضار (مثال: عصير البندورة) |  |  |  |  |  |  | نصف كوب |  |  |  | |
